# The Diagnosis of Small-Pox

**Published:** 1902-02-08

**Authors:** 


### THE DIAGNOSIS OF SMALL-POX.

The present is a time at which we may usefully call attention to the serious difficulties which may sometimes arise in practice with reference to the question of diagnosis of small-pox in its early stages. It is unquestionably true that large numbers of medical practitioners have never seen a case of small-pox, and that their knowledge of its aspects rests upon descriptions which are sufficiently likely to be based upon the more severe and more pronounced examples of the disease. At the same time, the existence in our midst of large numbers of adults who are only imperfectly protected by vaccination, who have perhaps been vaccinated in a single place in infancy, and never revaccinated, and who are presumably liable to contract a form of small-pox which may be modified favourably alike in its appearance and in its course, but which is capable of communicating it in its severest form to the wholly susceptible constitutes a condition which has so far modified the problem as to afford cases of eruptive fever which fail to correspond entirely with the typical form of variola, and may therefore easily pass unrecognised, while at the same time they are sources of continual danger to the public, and of danger heightened by the precise proportion in which it is liable to escape recognition. Many of these cases resemble chicken-pox, and, in some of them, it may be impossible to pronounce upon their precise character until time has been allowed for the complete development of the eruption. Coincidently with the prevalence of small-pox, undoubted chicken-pox has shown itself in several localities, and has supplied an additional complication to an already difficult problem, for a family practitioner would be likely, as a matter of course, to keep a guard over his tongue as long as he was in any doubt as between the mild and the severe disease.

In these circumstances the thoughts both of medical practitioners and of parents have not unnaturally turned towards the Medical Officers of Health themselves, and a very general feeling has been expressed that their services as diagnosticians ought, in all doubtful cases, to be placed at the disposal of the public and of the profession. However natural and however general, this feeling is none the less unreasonable. There would, in the first place, be no evidence of the competency of the officials in question to bear the responsibility that would be cast upon them. There is no assurance that a medical officer of health has ever seen a case of small-pox. His public health diploma implies that he has attended the practice of a hospital for infectious disease, but it affords no special proof as to his experience either of small-pox or of any other malady. In the next place, the duties of his position require him to be easily accessible in some given place, and are wholly incompatible with a demand that he should be at the beck and call of private practitioners, and be liable to be summoned to all parts of his district by urgent calls to visit patients supposed to be suffering from small-pox, and concerning whom the question of notification was to remain unsettled until his arrival. It is manifest that some other way of escape from the difficulty is desirable. The London County Council has taken a decided step by requiring, as a temporary measure, that cases of chicken-pox shall be notified. This will, of course, do something, and may perhaps do much, to remove the burden of doubt which has hitherto oppressed practitioners; but, at the same time, it will inflict unnecessary hardship upon a certain number of families. Some municipalities have boldly grappled with the question, and have retained the services of medical men within their boundaries who are well acquainted with small-pox, and have made them available as consultants to any who may be in doubt, the authorities making provision for their remuneration. Here, probably, is the best solution of the problem. It is curiously characteristic of London ways that of the various public bodies by whom we are governed—all of which are elected by the same people and draw out of the same long-suffering pockets—some should be earnestly engaged in rectifying evils which others have been equally earnestly endeavouring to perpetuate. The metropolis is indebted for its small-pox primarily, of course, to the Government and the Legislature; but secondarily, and in no small degree, to the neglect of locally-elected authorities to put into force even such remnants of power for the enforcement of vaccination as they possessed. Alike in their elections of the School Board and of the Boards of Guardians, Londoners have sown the wind; and it would be only fair that their lords of misrule should take a full share of the difficulties incidental to the harvest. Unfortunately, these fall upon other branches of local administration.

